# Supplementary material for: A high-resolution protein-RNA binding atlas for structure-aware interaction prediction and post-transcriptional regulatory modeling
Source: bioRxiv. 2026 Oct 29:2026.10.12.758580. Preprint. [Version 2] doi: 10.64898/2026.10.12.758580 (PMC13733719; doi:10.64898/2026.10.12.758580)
Supplement: Supplementary file 1 [file NIHPP2026.10.12.758580v2-supplement-1.pdf]

bioRxiv preprint doi: https://doi.org/10.64898/2026.10.12.758580; this version posted October 29, 2026. The copyright holder for this preprint (which was not certified by peer review) is the author/funder, who has granted bioRxiv a license to display the preprint in perpetuity. It is made available under a CC-BY 4.0 International license.

A high-resolution protein-RNA binding atlas for structure-aware interaction prediction and post-transcriptional regulatory modeling

Supplementary Material

A Acknowledgements 2

B Funding 2

C Data, code, and materials availability 2

D Supplementary figures 3

Figure S1. Optimization and validation of the eclair CLIP workflow. 3

Figure S2. Extended ecCLIP quality metrics and Borzuki validation. 5

Figure S3. Performance of AI models in predicting RBP binding. 6

Figure S4. Extended MPRC support for Pythia-nominated structure-dependent RBP binding. 7

Figure S5. Predicted-effect stratification and high-leverage SNPs for RBP-specific Pythia variant-effect models. 8

bioRxiv preprint doi: https://doi.org/10.64898/2026.10.12.758580; this version posted October 29, 2026. The copyright holder for this preprint (which was not certified by peer review) is the author/funder, who has granted bioRxiv a license to display the preprint in perpetuity. It is made available under a CC-BY 4.0 International license.

A. Acknowledgements

We thank A. Pawluk and C. Ricci-Tam for reading earlier versions of this manuscript and helping us with the content. We thank Albertas Navickas for input on eclair CLIP method development.

B. Funding

L.F. was a Zena Werb Scholar supported by T32CA108462. H.K. was a Zena Werb Scholar supported by T32CA108462. Sequencing was performed at the UCSF Center for Advanced Technology (CAT), supported by UCSF PBBR, RRP IMIA, and NIH 1S10OD028511-01 grants. H.S.N. is supported by the Canadian Institutes of Health Research (CIHR) grant PJT-173317 and resource allocations from Digital Research Alliance of Canada, and holds a CIHR Canada Research Chair. E.M.S. and M.D.L. are supported by Biohub. H.G. is a Core Investigator at Arc Institute, and this research was supported by Arc.

C. Data, code, and materials availability

All data and code needed to evaluate and reproduce the results in the paper are present in the paper and/or the Supplementary Materials.

- Pythia codebase is available at `https://github.com/goodarzilab/pythia`.
- Pre-trained Borzuki model weights and code are available at `https://github.com/calico/borzoi-paper/tree/main/extensions/borzuki`.
- All raw and processed sequencing data are available from the Gene Expression Omnibus (GEO) under accession number GSE349934.

bioRxiv preprint doi: https://doi.org/10.64898/2026.10.12.758580; this version posted October 29, 2026. The copyright holder for this preprint (which was not certified by peer review) is the author/funder, who has granted bioRxiv a license to display the preprint in perpetuity. It is made available under a CC-BY 4.0 International license.

D. Supplementary figures

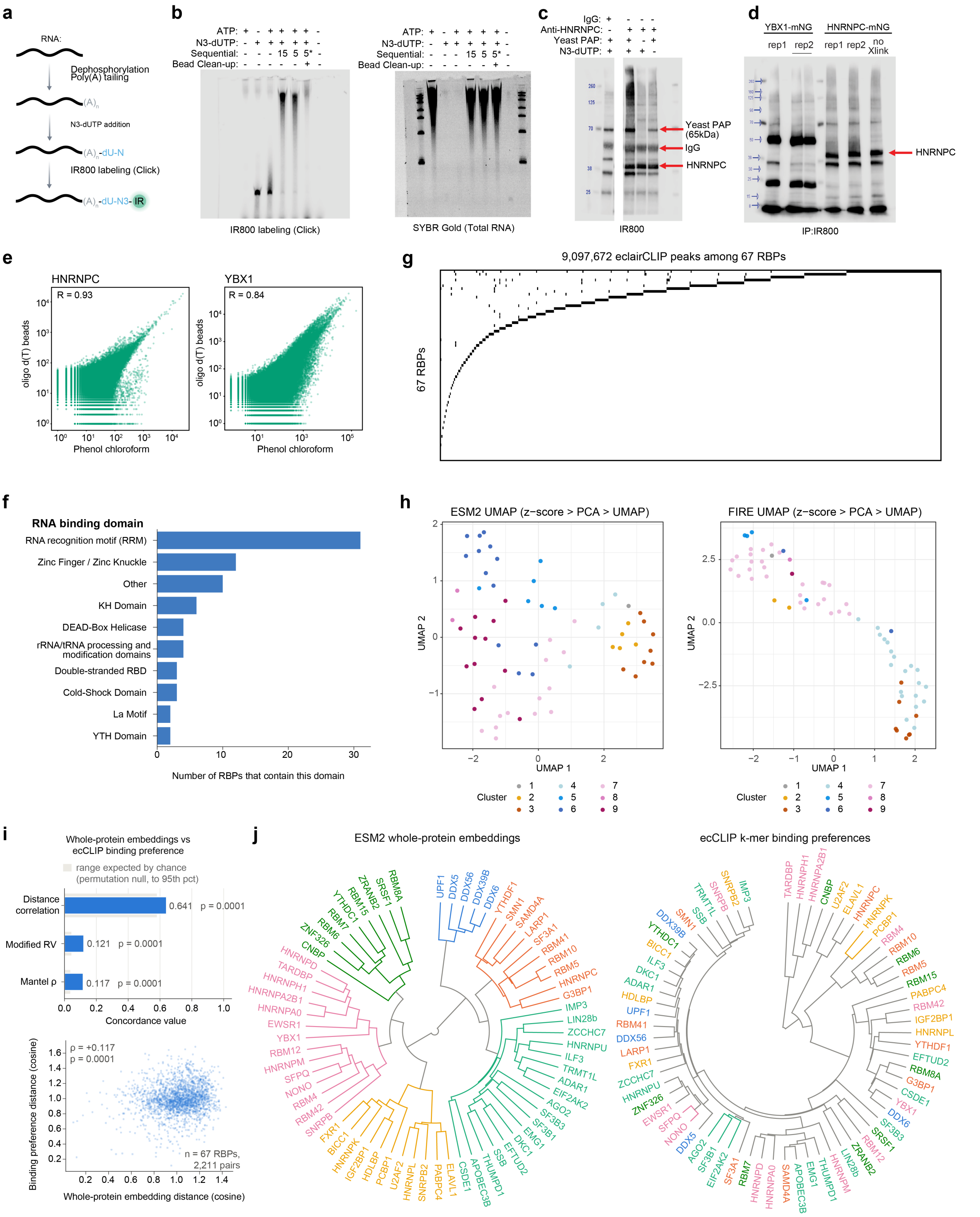

Figure S1 | **Optimization and validation of the eclair CLIP workflow.** Legend continued on the next page.

bioRxiv preprint doi: https://doi.org/10.64898/2026.10.12.758580; this version posted October 29, 2026. The copyright holder for this preprint (which was not certified by peer review) is the author/funder, who has granted bioRxiv a license to display the preprint in perpetuity. It is made available under a CC-BY 4.0 International license.

Figure S1 | **Optimization and validation of the eclair CLIP workflow.** **(a)** Schematic of eclair CLIP RNA labeling strategy. **(b)** An RNA oligo was subjected to poly(A) tailing with Yeast PAP, N_3-dUTP azide incorporation, and IR800 click labeling. Denaturing PAGE comparing labeling efficiency across conditions varying ATP, N_3-dUTP concentration, sequence and timing of steps, and bead cleanup, visualized by IR800 (left) and SYBR Gold stain (right). Sequential (-) indicates co-addition of ATP and N_3-dUTP. Sequential (+) indicates ATP addition followed by N_3-dUTP addition with the number indicating time in minutes of N_3-dUTP incubation. Sequential* indicates ATP addition followed by SPRI clean-up followed by N_3-dUTP incubation for 5 minutes. **(c)** Blot showing immunoprecipitation of HNRNPC from UV-crosslinked HEK293T cells using an anti-HNRNPC antibody or anti-mouse IgG. Shown are IgG control compared to anti-HNRNPC pulldown with and without yeast PAP-mediated poly(A) tailing and N_3-dUTP labeling. All labeled with DBCO-IR800. Bands corresponding to yeast PAP (65 kDa), IgG, and HNRNPC are indicated. **(d)** Blot showing immunoprecipitation of split mNeonGreen-tagged RBPs (YBX1 and HNRNPC) from HEK293T cells. A neon green nanobody was used to immunoprecipitate and the ecCLIP workflow used to label the complexes. A no-crosslink control is included. **(e)** Comparison of RNA isolation methods for eclair CLIP. Scatter plots show per-gene read counts for RNA isolated by oligo d(T) magnetic beads versus phenol chloroform extraction for HNRNPC (Pearson R = 0.93) and YBX1 (Pearson R = 0.84), demonstrating high concordance between methods. **(f)** Distribution of RNA binding domains among proteins in the ecCLIP dataset. Some proteins contain multiple RNA binding domain types. **(g)** Distribution of ecCLIP peaks across 67 RBPs. Dot plot shows the number of peaks called per RBP, ranked by peak count. A total of 9,097,672 peaks were identified across all 67 RBPs. **(h)** UMAP projections of ESM2 protein embeddings (left) and ecCLIP-derived sequence preferences labeled FIRE (right), following z-score normalization and principal component analysis. Points represent RBPs and are colored by clusters within each representation. **(i)** Whole-protein version of the analysis in Fig. 1c: concordance statistics between ESM2 whole-protein embeddings and ecCLIP-derived binding preferences, shown against the permutation-null range (grey bands), with the corresponding pairwise cosine-distance scatter (2,211 RBP pairs). **(j)** Circular dendrograms as in Fig. 1d, built from whole-protein embeddings (left) and ecCLIP-derived binding preferences (right), both colored by the protein-side grouping.

bioRxiv preprint doi: https://doi.org/10.64898/2026.10.12.758580; this version posted October 29, 2026. The copyright holder for this preprint (which was not certified by peer review) is the author/funder, who has granted bioRxiv a license to display the preprint in perpetuity. It is made available under a CC-BY 4.0 International license.

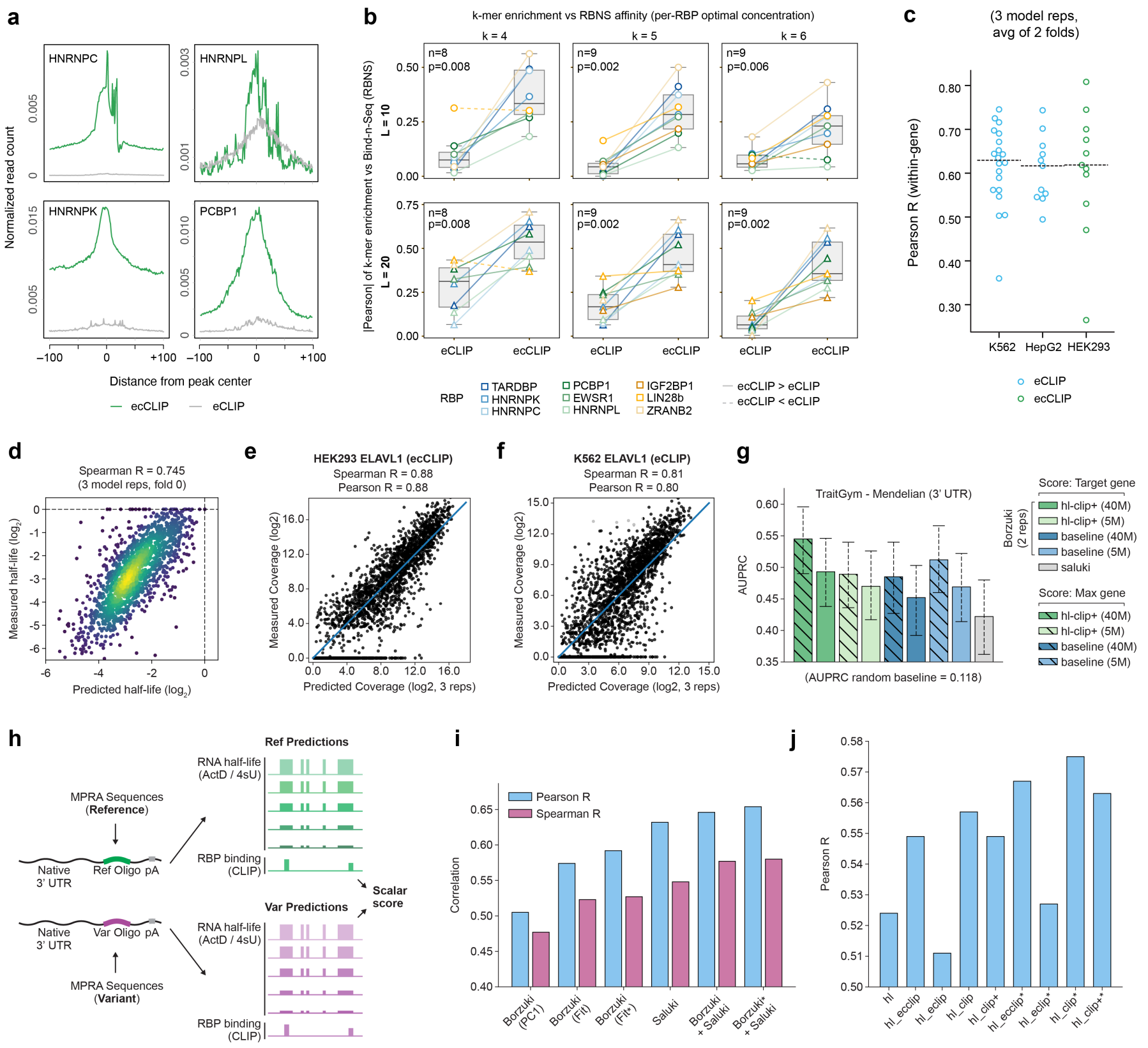

Figure S2 | **Extended ecCLIP quality metrics and Borzuki validation.** **(a)** Additional aggregate profiles comparing ecCLIP and eCLIP read signal centered on peak summits for matched RBPs. **(b)** Robustness of k-mer concordance to RBNS concentration choice. The absolute Pearson correlation between CLIP-derived k-mer enrichment and RBNS affinity is shown using the optimal RBNS protein concentration for each RBP, rather than the fixed 1300 nM concentration used in Fig. 2c. Results are shown for k = 4, 5, and 6 and peak-centered windows of 10 or 20 nucleotides. Lines connect matched RBPs between eCLIP and ecCLIP; solid lines indicate stronger concordance for ecCLIP and dashed lines indicate stronger concordance for eCLIP. P-values correspond to paired comparisons between eCLIP and ecCLIP. **(c)** Test set Pearson R when predicting CLIP-seq profiles at bin-level resolution within genes for held-out test data, shown for both ecCLIP and ENCODE eCLIP tracks. **(d)** Predicted versus measured mRNA degradation rates derived from a K562 actinomycin D transcriptional shutoff experiment. Predicted half-lives were derived by fitting exponential decay curves to Borzuki-predicted exon-aggregated coverage across time points. Spearman R = 0.745 with experimental measurements; Spearman R = 0.728 with Saluki's cell type-agnostic PC1 half-life summary. **(e)** Predicted versus measured gene-level exon-aggregated log-coverage for the ELAVL1 CLIP-seq experiment in the ecCLIP dataset. **(f)** Predicted versus measured gene-level exon-aggregated log-coverage for an ENCODE eCLIP ELAVL1 experiment. **(g)** Comparison between Saluki and Borzuki when classifying putative causal 3′ UTR variants associated with Mendelian traits from TraitGym (Benegas et al., 2025). Variant scoring is compared using the annotated target gene or the gene context with the largest predicted effect. **(h)** Illustration of variant scoring procedure when predicting mRNA half-life effects measured in an MPRA experiment in Beas2B cells (Siegel et al., 2022). Reference and variant oligos are inserted in endogenous 3′ UTRs and their predicted effect on RNA half-life and CLIP binding is calculated using Borzuki. For each RNA-seq or CLIP-seq track predicted by the model, the total log fold-change in coverage across the gene's exons due to the mutation is calculated. These functional multi-track scores are combined into a scalar variant impact using a secondary regressor model, which is fitted either on reference measurements from the MPRA assay or PCA values from the Saluki model's training data (Methods). **(i)** Comparison between Saluki and Borzuki at the task of predicting half-life MPRA variant effects (’PC1’ / ’Fit’ = Borzuki's multi-track scores are either fit to Saluki's PC1 measurements or to the MPRA's reference values, ‘*’ = variant scores are derived from CLIP-seq tracks in addition to half-life RNA-seq tracks). **(j)** Correlation metrics when predicting MPRA-derived half-life variant effects using versions of Borzuki models trained on subsets of training data (‘hl’ = trained on base ENCODE/FANTOM5 DNase/RNA-seq/CAGE tracks and RNA half-life time-course tracks, ‘hl_ecclip’ = additionally trained on ecCLIP experiments, ‘hl_eclip’ = additionally trained on ENCODE eCLIP, ‘hl_clip’ = trained on ecCLIP and eCLIP tracks, ‘hl_clip+’ = trained on all CLIP-seq experiments and polyadenylation Perturb-seq. ‘*’ = variant scores are derived from CLIP-seq tracks in addition to half-life RNA-seq tracks).

bioRxiv preprint doi: https://doi.org/10.64898/2026.10.12.758580; this version posted October 29, 2026. The copyright holder for this preprint (which was not certified by peer review) is the author/funder, who has granted bioRxiv a license to display the preprint in perpetuity. It is made available under a CC-BY 4.0 International license.

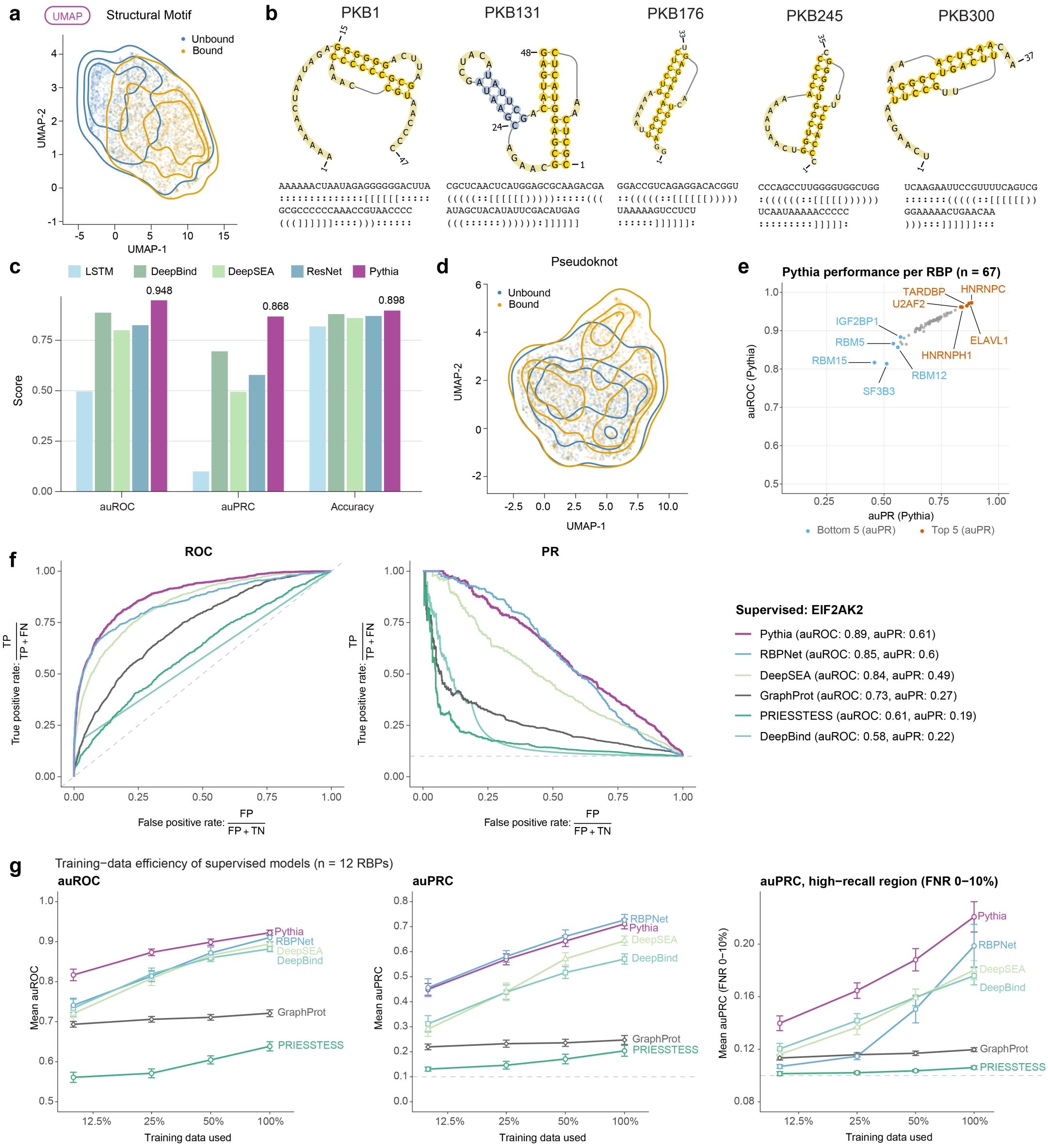

Figure S3 | **Performance of AI models in predicting RBP binding.** **(a)** Generalization to sequence-independent motifs. UMAP projection of Pythia embeddings for held-out synthetic sequences with or without the CFG-defined structural motif. The two groups overlap; this visualization alone does not establish motif classification accuracy. **(b)** Pseudoknot structure prediction. We performed in silico mutations on five distinct pseudoknot structures, generating positive examples that preserved base-pairings and negative examples that disrupted them. **(c)** Pythia distinguished preserved from disrupted pseudoknot structures with an AUROC = 0.95 and AUPRC = 0.87, outperforming other models (LSTM, DeepBind, DeepSEA, ResNet) and demonstrating structural sensitivity. **(d)** Latent space organization. UMAP projection of Pythia embeddings for preserved and disrupted pseudoknot examples. The overlapping distributions are shown descriptively; classification performance is quantified in (c). **(e)** Scatterplot of Pythia auPR and auROC performance per RBP. **(f)** ROC (left) and precision-recall (PR) curves for supervised models predicting genome-wide binding of EIF2AK2. The y-axis of the ROC plot shows true positive rate [TP/(TP+FN)] and the x-axis shows false positive rate [FP/(FP+TN)]. The y-axis of the PR plot shows precision [TP/(TP+FP)] and the x-axis shows recall [TP/(TP+FN)]. Pythia (purple; auROC = 0.89, auPR = 0.61) outperforms RBPNet (auROC = 0.85, auPR = 0.60), DeepSEA (auROC = 0.84, auPR = 0.49), GraphProt (auROC = 0.73, auPR = 0.27), PRIESSTESS (auROC = 0.61, auPR = 0.19), and DeepBind (auROC = 0.58, auPR = 0.22). **(g)** Training-data efficiency of supervised models. Models were trained on 10, 25, 50 or 100% of each RBP's training set (stratified subsamples, identical across models; three seeds) for the 12 RBPs in **Fig. 3d** and evaluated on the same 10,000 validation sequences. Left, mean auROC; middle, mean auPRC; right, mean auPRC in the high-recall region (recall 0.9–1). Error bars, s.e.m. across RBPs.

bioRxiv preprint doi: https://doi.org/10.64898/2026.10.12.758580; this version posted October 29, 2026. The copyright holder for this preprint (which was not certified by peer review) is the author/funder, who has granted bioRxiv a license to display the preprint in perpetuity. It is made available under a CC-BY 4.0 International license.

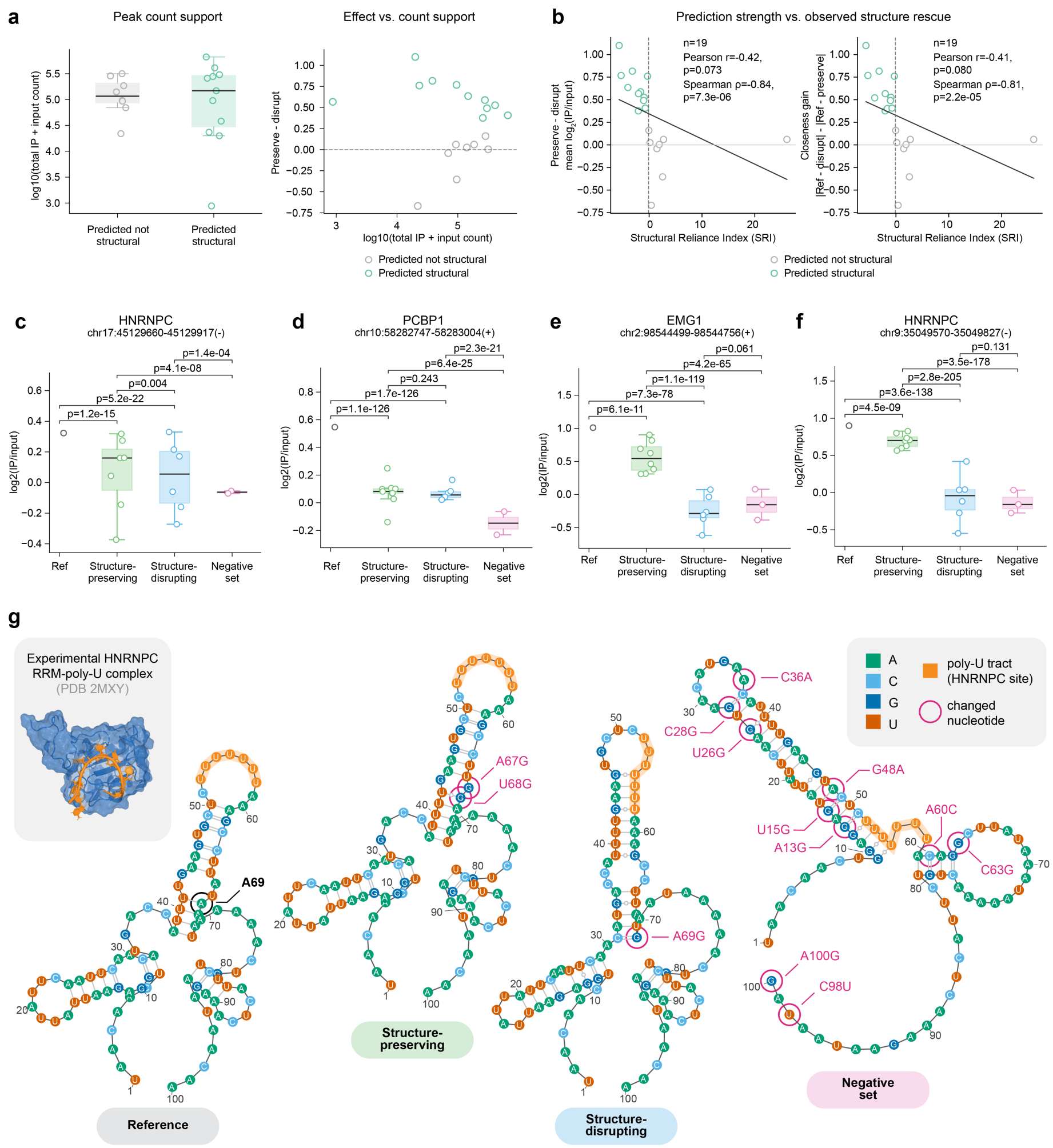

Figure S4 | **Extended MPRC support for Pythia-nominated structure-dependent RBP binding.** **(a)** Count support for structure-rescue summaries. Each point is one reference-enriched peak with Ref $\log_2$(IP/input) > 0.25. The left panel compares total peak counts, computed as summed IP plus input counts across constructs, between predicted not-structure-dependent and predicted structure-dependent groups on a $\log_{10}$ scale. The right panel plots the preserve-minus-disrupt rescue metric against total peak count to test whether observed rescue effects are concentrated in low- or high-count peaks. Points are colored by predicted structural class. **(b)** Pythia prediction strength versus observed structure-rescue metrics. Each point is one reference-enriched peak with Ref $\log_2$(IP/input) > 0.25. The x-axis shows a Structural Reliance Index (SRI), defined per peak as log ratio of sequence-arm to structure-arm DeepLIFT attribution under a zero-baseline reference, and the dashed vertical line marks the prediction threshold of −0.1 used to define predicted structure-dependent peaks. The left panel shows preserve-minus-disrupt, defined as mean structure-preserving enrichment minus mean structure-disrupting enrichment. The right panel shows closeness gain, defined as abs(Ref - Structure-disrupting) - abs(Ref - Structure-preserving). Positive y-values indicate a stronger structure-preserving rescue pattern. Points are colored by threshold-defined prediction group; fitted lines and Pearson/Spearman statistics summarize continuous-score associations. **(c–f)** Additional representative structure-variant validation examples for HNRNPC at chr17:45,129,660-45,129,917(-), PCBP1 at chr10:58,282,747-58,283,004(+), EMG1 at chr2:98,544,499-98,544,756(+), and HNRNPC at chr9:35,049,570-35,049,827(-). Each point is one construct summarized across replicates as $\log_2$(IP/input). Constructs are grouped as reference, structure-preserving, structure-disrupting, or negative-control reporters. Boxes are shown for groups with multiple constructs, overlaid open points show individual construct values, and brackets report pairwise binomial logistic-regression P-values where valid. **(g)** Structural context for the HNRNPC validation example. The experimentally determined HNRNPC RRM-poly-U complex (PDB 2MXY; Cieniková et al., 2014; Cienikova et al., 2015) is shown alongside sequence-based secondary-structure predictions for reference, structure-preserving, structure-disrupting, and negative-control constructs. The poly-U tract is highlighted in orange and altered nucleotides are circled. RNA structures were predicted with ViennaRNA and displayed with VARNA (Methods; Lorenz et al., 2011; Darty et al., 2009). These predictions provide structural context and are not direct measurements of reporter folding in cells.

bioRxiv preprint doi: https://doi.org/10.64898/2026.10.12.758580; this version posted October 29, 2026. The copyright holder for this preprint (which was not certified by peer review) is the author/funder, who has granted bioRxiv a license to display the preprint in perpetuity. It is made available under a CC-BY 4.0 International license.

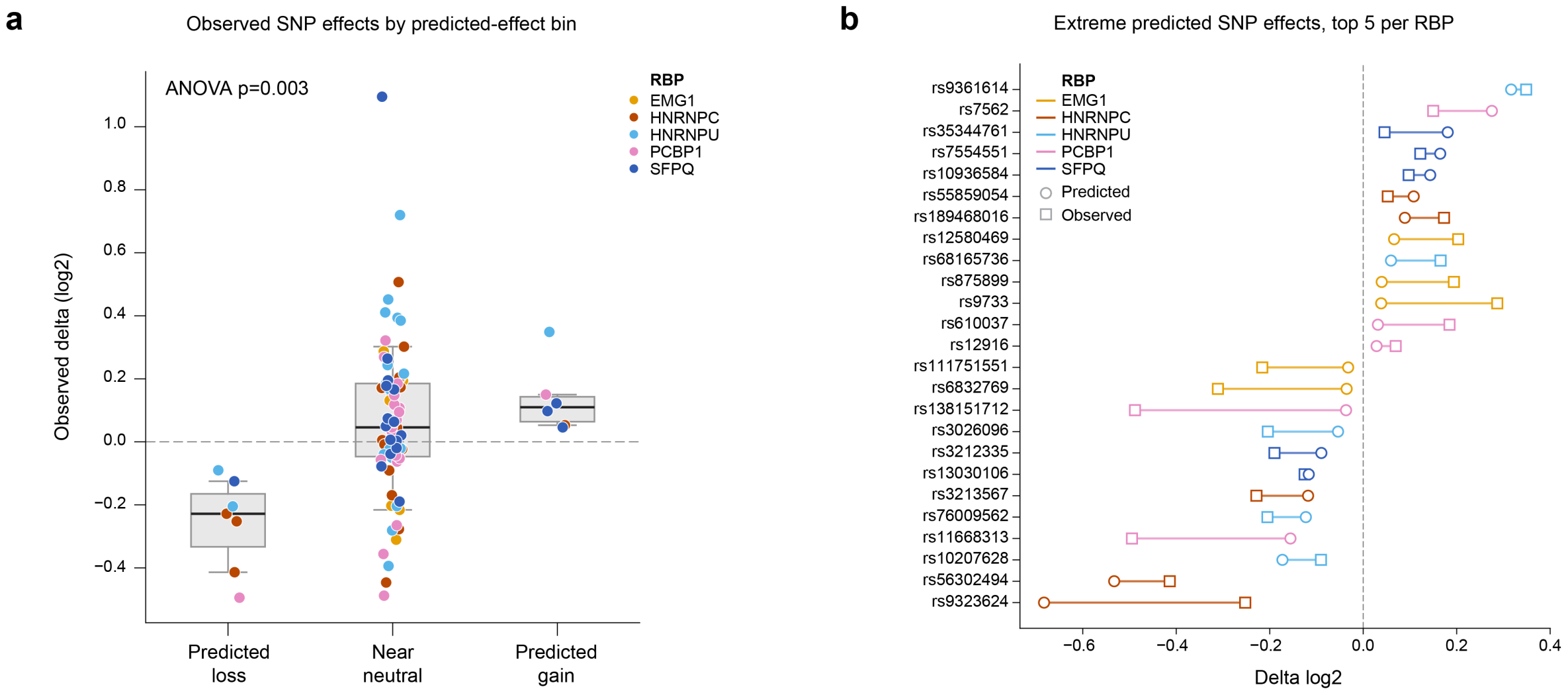

Figure S5 | **Predicted-effect stratification and high-leverage SNPs for RBP-specific Pythia variant-effect models. (a)** Observed SNP effects grouped by predicted-effect bin. SNPs with valid, converged model fits were binned by Pythia predicted change into predicted loss (delta < -0.1), near neutral ($-0.1 \leq$ delta ≤ 0.1), and predicted gain (delta > 0.1). The y-axis shows the observed logistic-regression SNP effect in $\log_2$ units. Boxes summarize each bin, with the box spanning the 25th to 75th percentiles, the median line marking the 50th percentile, and whiskers set to the 10th and 90th percentiles. Overlaid points show individual SNPs colored by RBP. The dashed horizontal line marks zero observed effect. The text box reports the one-way ANOVA P-value across the three observed-delta distributions. **(b)** Extreme predicted SNP effects. For each RBP, the top five SNPs by absolute Pythia v3 predicted effect are shown. Each horizontal segment connects the predicted SNP effect (circle) to the observed logistic-regression effect (square), both in $\log_2$ units. The y-axis shows SNP names, and segments and markers are colored by RBP. The vertical dashed line marks zero effect. This plot highlights high-leverage predicted SNPs and shows whether the observed direction and magnitude agree with the prediction.
